# Defining new reference intervals for serum free light chains in individuals with chronic kidney disease: Results of the iStopMM study

**DOI:** 10.1038/s41408-022-00732-3

**Published:** 2022-09-14

**Authors:** Thorir Einarsson Long, Olafur Skuli Indridason, Runolfur Palsson, Sæmundur Rognvaldsson, Thorvardur Jon Love, Sigrun Thorsteinsdottir, Ingigerdur Solveig Sverrisdottir, Brynjar Vidarsson, Pall Torfi Onundarson, Bjarni Agnar Agnarsson, Margret Sigurdardottir, Ingunn Thorsteinsdottir, Isleifur Olafsson, Asdis Rosa Thordardottir, Elias Eythorsson, Asbjorn Jonsson, Gauti Gislason, Andri Olafsson, Hlif Steingrimsdottir, Malin Hultcrantz, Brian G. M. Durie, Stephen Harding, Ola Landgren, Sigurdur Yngvi Kristinsson

**Affiliations:** 1 University of Iceland, Reykjavik, Iceland; 2 Skane University Hospital, Lund, Sweden; 3 Landspitali–The National University Hospital of Iceland, Reykjavik, Iceland; 4 Department of Hematology, Rigshospitalet, Copenhagen, Denmark; 5 Sahlgrenska University Hospital, Gothenburg, Sweden; 6 Myeloma Service, Department of Medicine, Memorial Sloan Kettering Cancer Center, New York, NY USA; 7 Samuel Oschin Comprehensive Cancer Institute, Cedars-Sinai Outpatient Cancer Center, Los Angeles, CA USA; 8 Binding Site Group Ltd, Birmingham, UK; 9 Myeloma Program, Department of Medicine, University of Miami, Sylvester Comprehensive Cancer Center, Miami, USA

**Keywords:** Myeloma, Myeloma

## Abstract

Serum free light chain (FLC) concentration is greatly affected by kidney function. Using a large prospective population-based cohort, we aimed to establish a reference interval for FLCs in persons with chronic kidney disease (CKD). A total of 75422 participants of the iStopMM study were screened with serum FLC, serum protein electrophoresis and immunofixation. Estimated glomerular filtration rate (eGFR) was calculated from serum creatinine. Central 99% reference intervals were determined, and 95% confidence intervals calculated. Included were 6461 (12%) participants with measured FLCs, eGFR < 60 mL/min/1.73 m^2^, not receiving renal replacement therapy, and without evidence of monoclonality. Using current reference intervals, 60% and 21% had kappa and lambda FLC values outside the normal range. The FLC ratio was outside standard reference interval (0.26–1.65) in 9% of participants and outside current kidney reference interval (0.37–3.10) in 0.7%. New reference intervals for FLC and FLC ratio were established. New reference intervals for the FLC ratio were 0.46–2.62, 0.48–3.38, and 0.54–3.30 for eGFR 45–59, 30–44, and < 30 mL/min/1.73 m^2^ groups, respectively. The crude prevalence of LC-MGUS in CKD patients was 0.5%. We conclude that current reference intervals for FLC and FLC ratio are inaccurate in CKD patients and propose new eGFR based reference intervals to be implemented.

## Introduction

Monoclonal gammopathies include a wide spectrum of disorders ranging in severity from monoclonal gammopathy of undetermined significance (MGUS) and monoclonal gammopathy of renal significance (MGRS), to active multiple myeloma (MM), amyloid light-chain (AL) amyloidosis and non-Hodgkin lymphoma, among many others [1, 2]. MGUS is a precursor to MM and related diseases [3, 4], and light chain (LC)-MGUS a precursor to LC-MM and AL amyloidosis [5]. In addition to serum protein electrophoresis (SPEP) and serum immunofixation electrophoresis (IFE), the serum free light chain (FLC) assay has become the method of choice for detection, prognostication, and monitoring of monoclonal gammopathies [6]. LC-MGUS is defined as an FLC ratio and involved FLC level outside reference intervals without evidence of end-organ damage, MGUS, or other lymphoproliferative disorders (LP). However, as FLCs are primarily cleared by the kidneys, the kidney function is a major determinant of the serum concentration [7]. The prevalence of LC-MGUS in individuals with chronic kidney disease (CKD) is uncertain.

The reference intervals for serum kappa FLC (3.3–19.4 mg/L), lambda FLC (5.7–26.3 mg/L), and FLC ratio (0.26–1.65) were defined in a small cohort (*N* = 282) with normal kidney function [8]. A kidney reference interval for FLC ratio (0.37–3.10) for patients with estimated glomerular filtration rate (eGFR) < 60 mL/min/1.73 m^2^ based on a study of few individuals with CKD stage 3 and above [9]. The kidney reference interval for FLC ratio was later validated in a cohort of patients with MM on dialysis [10]. No validation study on FLC ratio has been performed in patients with earlier stages of CKD or with other monoclonal gammopathies. No reference intervals for absolute levels of kappa and lambda FLCs have been proposed in people with CKD stage 3 or higher. It is of major importance to identify individuals with true monoclonal gammopathy, and as the prevalence of both monoclonal gammopathies and CKD increase with age [11, 12], it is imperative that the FLC assay is accurate and usable in individuals with CKD.

The Iceland Screens, Treats or Prevents MM (iStopMM) study is a nationwide, prospective screening study were all individuals born in 1975 and earlier were invited for screening with SPEP and FLC with over 80,000 (54%) individuals providing informed consent [13]. Here we estimate the association between absolute FLCs levels and FLC ratio with kidney function in a large prospective population-based cohort. The aim was to establish a kidney reference interval for FLCs, and either confirm the previously reported kidney reference interval for the FLC ratio or propose new reference intervals for patients with CKD.

## Material and methods

### Ethical considerations

The study was approved by the National Bioethics Committee and the Data Protection Authority in Iceland. All participants enrolled in the study gave their informed written consent.

### Participants and data collection

The iStopMM study is a nationwide, prospective study with the overall aim to assess the benefits and harms of early detection by screening for MGUS and LC-MGUS (ClinicalTrials.gov number NCT03327597). All residents of Iceland registered on September 9th 2016, born in 1975 or earlier (*n* = 148,711) were invited to participate in the study, of whom 80,759 (54.3%) gave their written consent. A detailed description of the iStopMM study methods have previously been published [13].

Sera from 75,422 (93%) study participants were obtained and shipped to The Binding Site Ltd. in Birmingham, UK for analysis of SPEP, IFE, and sFLC. ﻿All samples were screened for M protein by SPEP using capillary zone electrophoresis (CZE; Helena Laboratories, Texas, USA). IFE (Helena Laboratories, TX, USA) was performed when the CZE was suspicious for M protein bands or when the FLC results based on standard reference intervals were abnormal. Quantification of kappa and lambda FLCs was done using FREELITE^TM^ reagents sets from The Binding Site Ltd. A more detailed description of analytic methods have been published elsewhere [13].

All serum creatinine (SCr) measurements from the participants performed for clinical indications were collected from a central laboratory database. The SCr values were used to calculate eGFR using the Chronic Kidney Disease Epidemiology Collaboration (CKD-EPI) equation [14]. Demographic data and all previous International Classification of Diseases, 9th and 10th revisions (ICD-9 and ICD-10) diagnosis codes were obtained from the Icelandic Directorate of Health for assessment of the participants‘ comorbid conditions, categorized into disease groups for analysis [13]. The accuracy of disease diagnosis in the registry has recently been validated [15]. Information on renal replacement therapy (RRT), i.e. hemodialysis, peritoneal dialysis, and kidney transplantation, was obtained from the Icelandic End-Stage Kidney Disease Registry.

Participants with M protein on IFE or involved/uninvolved sFLC ratio ≥100 with serum involved FLC ≥ 100 mg/L (myeloma defining event) [16], were excluded. The involved FLC was defined as the FLC with the higher absolute concentration. The SCr measurement closest to the screening was used for calculation of eGFR; participants without SCr measurement available within one year before or after screening were excluded. An algorithm based partly on the KDIGO diagnostic criteria for acute kidney injury (AKI) [17], that has been validated [18], was used to detect SCr measurements consistent with an AKI episode. These SCr measurements were excluded, alongside any SCr measurements performed in the ten day period following presumed AKI. This was done to minimize the effect of sudden changes in kidney function on our findings. Participants who were undergoing hemodialysis or peritoneal dialysis or had a transplanted kidney at the time of screening were excluded from main analysis and analyzed seperately. LC-MGUS was defined as an FLC ratio outside the reference interval and an increase in involved FLC without evidence of monoclonal heavy chain on SPEP or IFE, or end-organ damage attributed to the plasma cell proliferative disorder [5].

### Statistical analysis and data configuration

Normality of data was assessed by direct visualization and the Anderson-Darling test [19]. The 2.5 and 97.5 percentiles of the distributions of kappa FLC, lambda FLC, and FLC ratio were assessed in the whole group, and in subgroups based on age, sex and eGFR representing CKD stages 3–5. A nonparametric bootstrapping method was used to calculate the 95% confidence intervals (CI) for the central 95 and 99 percentile limits. Outliers were assessed using Horn’s algorithm [20] but were not removed from analysis.

The decision on whether a reference interval was partitioned was based on distribution of subgroups (age, sex, eGFR) outside whole group reference interval. If a subgroup distribution outside central 95% reference interval exceeded 4.1% or was below 0.9%, the reference interval was partitioned based on subgroups as recommended by Lahti et al. [21]. A minimum sample size of 120 individuals was used to determine a subgroup reference interval as recommended by joint guidelines from the Clinical and Laboratory Standards Institute and the International Federation of Clinical Chemistry [22]. A sample size of approximately 340 should give an estimate of the population mean with an precision of + /−2%. Central 95% and 99% reference intervals were estimated for serum kappa and lambda FLC, and the FLC ratio for the whole study cohort, and subgroups of age, sex, and eGFR (< 60 mL/min/1.73 m^2^). A substantial proportion of eGFR subgroup distribution was outside the whole group common reference limits (Supplementary Table IABC). Accordingly, the reference intervals were defined by eGFR categories 45–59, 30–44, and < 30 mL/min/1.73 m^2^ for kappa and lambda FLC, and the FLC ratio. A considerable proportion of participants with eGFR < 15 mL/min/1.73 m^2^ had values outside the common reference interval, but due to their low number (*N* = 55), the reference intervals for eGFR < 15 and 15–29 mL/min/1.73 m^2^ were combined. After partitioning by these eGFR categories (45–59, 30–44, and < 30 mL/min/1.73 m^2^), the proportion of age and sex subgroups with levels outside reference intervals were considered appropriate. A sensitivity analysis using only participants with SCr measured at the time of screening was performed.

The revised reference intervals were plotted and compared to currently used reference intervals as well as previously published kidney reference interval of FLC ratio for individuals with reduced kidney function. Correlation between FLC measurements, eGFR and age was assessed graphically and using the Spearman correlation coefficient. Comparison of baseline characteristics and comorbid conditions between groups was carried out using the Chi-square test, the t-test/Wilcoxon rank sum test, analysis of variance/Kruskal– Wallis test, as appropriate.

The prevalence of LC-MGUS in individuals with CKD was assessed both using previously published reference intervals and our proposed reference intervals and the crude rate reported. All statistical analysis and data visualization was performed using R (version 3.6.3).

## Results

**Fig. 1:**
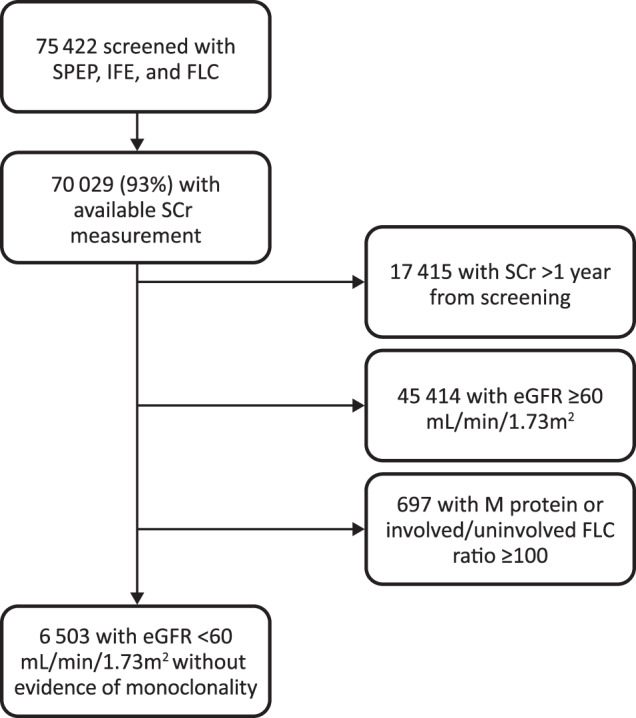
Flowchart outlining the study population and excluded participants. SPEP Serum protein electrophoresis, IFE Immunofixation, FLC Free light chains, SCr Serum creatinine, eGFR Estimated glomerular filtration rate.

A total of 75,422 participants in the iStopMM study were screened with SPEP, IFE, and FLC. After application of exclusion criteria, 6503 (12%) participants with eGFR < 60 mL/min/1.73 m^2^ and without evidence of heavy chain MGUS were included (Fig. 1). Ten (0.2%) participants were on dialysis and 32 (0.5%) had a functioning kidney transplant at the time of screening and both groups were analyzed seperately. The baseline characteristics of the study cohort are demonstrated in Table 1.

**Table 1:** Baseline characteristics of iStopMM participants with chronic kidney disease stages 3–5, stratified by estimated glomerular filtration rate at the time of screening.

	Overall	< 30	30–44	45–59	Dialysis	Transplant	*p*
		mL/min/1.73 m^2^	mL/min/1.73 m^2^	mL/min/1.73 m^2^			
*n*	6503	384	1465	4612	10	32	
Age, years (median [IQR])	74 (68–81)	79 (74–85)	78 (72–84)	73 (66–79)	71 (65–77)	67 (59–69)	<0.001
Male (%)	2913 (44.8)	194 (50.5)	649 (44.3)	2044 (44.3)	7 (70.0)	19 (59.4)	0.027
Serum creatinine, μmol/L (median [IQR])	109 (94–125)	196 (166–239)	129 (114–144)	101 (91–114)	..	124 (115–160)	<0.001
eGFR, mL/min/1.73 m^2^ (median [IQR])	51 [43–56]	25 (19–28)	40 (36–43)	55 (51–57)	..	44 (37–53)	<0.001
Free kappa, mg/L (median [IQR])	21.7 (16.6–29.4)	41.5 (31.8–59.3)	27.2 (20.8–36.4)	19.5 (15.5–25.2)	89.0 (67.2–137.2)	26.1 (17.7–30.9)	<0.001
Free lambda, mg/L (median [IQR])	19.0 (14.8–25.0)	33.1 [24.1–46.1)	22.6 [17.8–29.1)	17.5 (14.0–22.1)	66.9 (57.9–91.6]	22.1 (17.1–27.2)	<0.001
FLC ratio (median [IQR])	1.16 (0.97–1.39)	1.31 (1.10–1.57)	1.21 (1.01–1.45)	1.13 (0.94–1.35)	1.27 (1.22–1.49)	1.15 (0.81–1.34)	<0.001
Screening to serum creatinine <24 h	4658 (71.6)	298 (77.6)	1054 (71.9)	3270 (70.9)	7 (70.0)	29 (90.6)	0.008
Hypertension (%)	4443 (69.7)	326 (85.1)	1139 (79.3)	2948 (65.3)	6 (60.0)	24 (75.0)	<0.001
Cardiac arrythmia (%)	1670 (26.2)	141 (36.8)	475 (33.1)	1042 (23.1)	4 (40.0)	8 (25.0)	<0.001
Ischemic heart disease (%)	1873 (29.4)	152 (39.7)	504 (35.1)	1202 (26.6)	4 (40.0)	11 (34.4)	<0.001
Peripheral artery disease (%)	643 (10.1)	66 (17.2)	192 (13.4)	376 (8.3)	3 (30.0)	6 (18.8)	<0.001
Heart failure (%)	934 (14.6)	138 (36.0)	318 (22.1)	466 (10.3)	2 (20.0)	10 (31.2)	<0.001
Obesity (%)	758 (11.9)	59 (15.4)	186 (13.0)	507 (11.2)	1 (10.0)	5 (15.6)	0.077
Diabetes (%)	1027 (16.1)	126 (32.9)	309 (21.5)	583 (12.9)	4 (40.0)	5 (15.6)	<0.001
Endocrine disorder (non-diabetic) (%)	1342 (21.0)	88 (23.0)	338 (23.5)	906 (20.1)	4 (40.0)	6 (18.8)	0.025
Chronic lung disease (%)	2481 (38.9)	169 (44.1)	587 (40.9)	1716 (38.0)	2 (20.0)	7 (21.9)	0.008
Liver disease (%)	178 (2.8)	11 (2.9)	43 (3.0)	123 (2.7)	0 (0.0)	1 (3.1)	0.962
Malignancy (%)	1469 (23.0)	127 (33.2)	397 (27.6)	938 (20.8)	4 (40.0)	3 (9.4)	<0.001
Neurological disease (%)	381 (6.0)	31 (8.1)	97 (6.8)	243 (5.4)	5 (50.0)	5 (15.6)	<0.001
Rheumatologic disease (%)	659 (10.3)	43 (11.2)	173 (12.0)	441 (9.8)	0 (0.0)	2 (6.2)	0.085

Data reported as median (interquartile range) or *N* (%) as appropriate. Demographic data and all previous International Classification of Diseases, 9th and 10th revisions (ICD-9 and ICD-10) diagnosis codes were obtained from the Icelandic Directorate of Health for assessment of the participants‘ comorbid conditions, categorized into disease groups for analysis [13]. The accuracy of disease diagnosis in the registry has recently been validated [15].

*eGFR* Estimated glomerular filtration rate, *FLC* Free light chain, *IQR* Interquartile range.

**Fig. 2:**
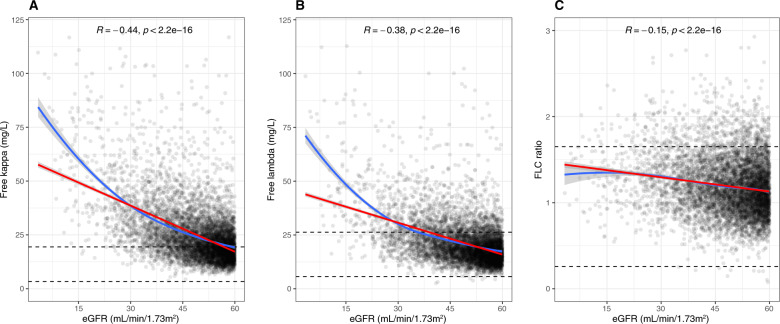
Demonstration of the correlation between serum free light chains and kidney function. Correlation between kappa FLC (**A**), lambda FLC (**B**), and FLC (**C**) ratio and eGFR. Red line demonstrates linear correlation and blue line local regression (loess). The standard reference intervals for kappa FLC (**A**), lambda FLC (**B**) and FLC ratio (**C**) are demonstrated with dashed lines. The y axis for kappa FLC and lambda FLC are truncated at 125 mg/L and FLC ratio at 3.

Among the 6503 participants, the median (interquartile range [IQR]) kappa FLC level was 21.7 mg/L (16.6–29.4), lambda FLC level was 19.0 mg/L (14.8–25.0), and the FLC ratio was 1.16 (0.97–1.39). Median (IQR) of serum kappa and lambda FLC levels, and the FLC ratio stratified by age, sex, and eGFR is shown in Table 2. Serum FLCs increased with declining eGFR, with a negative correlation between eGFR and serum kappa FLC (ρ = −0.44, *p* < 0.001) and lambda FLC (ρ = −0.38, *p* < 0.001) levels, and the FLC ratio (ρ = −0.15, *p* < 0.001) (Fig. 2A–C). Furthermore, older age was associated with increasing FLCs with a positive correlation between age and kappa FLC (ρ = 0.28, *p* < 0.001), lambda FLC (ρ = 0.19, *p* < 0.001), and FLC ratio (ρ = 0.18, *p* < 0.001). However, when stratified by eGFR categories this age-related increase in kappa and lambda FLC, and FLC ratio was substantially less pronounced although it remained statistically significant (Supplementary Figure 1A–C; Supplementary Table II). A weak but statistically significant positive correlation was noted between male sex and kappa FLC (ρ = 0.12, *p* < 0.001), lambda FLC (ρ = 0.09, *p* < 0.001), and the FLC ratio (ρ = 0.06, *p* < 0.001).

**Table 2:** Median (IQR) levels of serum kappa and lambda FLC and FLC ratio in subgroups according to age, sex, and categories of reduced estimated glomerular filtration rate.

	Kappa (mg/L)	Lambda (mg/L)	FLC ratio
Age (years)			
< 50	16.2 (13.6–25.5)	16.6 (12.7–23.0)	1.05 (0.87–1.28)
50–59	17.9 (13.5–25.1)	16.2 (12.8–22.9)	1.10 (0.91–1.30)
60–69	19.1 (14.9–24.9)	17.7 (14.0–22.7)	1.09 (0.91–1.29)
70–79	21.7 (16.9–28.8)	18.8 (15.0–24.6)	1.16 (0.97–1.39)
≥ 80	24.8 (19.1–33.9)	20.8 (16.2–26.8)	1.23 (1.02–1.46)
Sex			
Male	23.1 (17.6–32.0)	19.8 (15.3–26.1)	1.19 (0.98–1.42)
Female	20.4 (15.9–27.4)	18.2 (14.5–23.8)	1.13 (0.95–1.36)
eGFR (mL/min/1.73 m^2^)			
45–59	19.5 (15.5–25.2)	17.5 (14.0–22.1)	1.13 (0.94–1.35)
30–44	27.2 (20.8–36.4)	22.6 (17.8–29.1)	1.21 (1.01–1.45)
15–29	39.1 (30.2–54.8)	30.8 (23.4–40.5)	1.31 (1.12–1.57)
< 15	71.5 (54.0–84.1)	49.1 (38.9–77.5)	1.33 (1.03–1.59)

*eGFR* Estimated glomerular filtration rate, *FLC* Free light chain.

### Current reference intervals for kappa, lambda, and FLC ratio

**Fig. 3:**
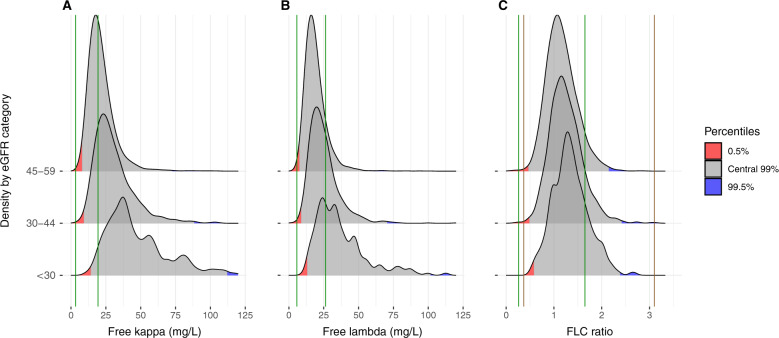
Comparison of the distribution of kappa and lambda free light chains (FLC) and FLC ratios to current reference intervals by estimated glomerular filtration rate (eGFR) subgroups. The portion of the distribution below the 0.5th percentile is shaded red and the portion above the 99.5th percentile is shaded blue. **A** Serum kappa FLC (green lines 3.3–19.4 mg/L). **B** Serum lambda FLC (green lines 5.7–26.3 mg/L). **C** FLC ratio (green lines – conventional reference interval: 0.26–1.65; brown lines – current kidney reference interval: 0.37–3.10). Kappa and lambda FLC are truncated at 120 mg/L and FLC ratio at 3.5 in the figures for better visualization. eGFR Estimated glomerular filtration rate, FLC Free light chains.

A well calibrated central 99% reference interval should partition an unselected sample from the target population such that 0.5% of persons lie below the lower limit and 0.5% above the upper limit. Using the current reference interval for serum kappa FLC (3.3–19.4 mg/L) among individuals with eGFR <60 mL/min/1.73 m^2^, 3890 (60%) participants had a kappa FLC level above the upper reference limit and no participant (0%) had a kappa FLC level below the lower reference limit (Fig. 3A). Similarly, using the current reference interval for serum lambda FLC (5.7–26.3 mg/L), 1375 (21%) participants had a lambda FLC level above the upper reference limit and only seven (0.1%) participants had a lambda FLC level below the lower reference limit (Fig. 3B). Finally, when the standard FLC ratio reference interval (0.26–1.65) was used, 597 (9%) participants had an FLC ratio above the upper reference limit and ten (0.1%) below the lower limit (Fig. 3C). Using the current kidney reference interval (0.37–3.10), 28 (0.4%) participants had an FLC ratio above the upper reference limit and 16 (0.3%) below the lower limit (Fig. 3C).

### Redefining FLC reference intervals for patients with chronic kidney disease

A single reference interval was constructed for all participants with eGFR <60 mL/min/1.73 m^2^ for serum kappa and lambda FLCs, and the FLC ratio. Using this reference interval would result in large proportions of participants in subgroups based on age, sex, and eGFR being diagnosed with light chain disease (abnormal FLC results; Supplementary Tables IA–C). The proportion of abnormal kappa and lambda FLC values would be highest in eGFR categories 15–29 and <15 mL/min/1.73 m^2^ with a rate of 14% and 55% for kappa FLC and 13% and 49% for lambda FLC, respectively. This whole-group reference interval for the FLC ratio was associated with lower rates of abnormal values with the highest rate of 7% in the eGFR 15–29 mL/min/1.73 m^2^ subgroup.

Because the whole-group reference interval resulted in an unreasonably high proportion of participants in the reduced eGFR subgroups being classified as having LC disease, the reference intervals were partitioned by eGFR categories. Due to few number of participants with eGFR < 15 mL/min/1.73 m^2^ (*N* = 55), a single combined reference interval was determined for eGFR < 15 and 15–29 mL/min/1.73 m^2^. The novel central 99% reference intervals for kappa FLC, lambda FLC, and FLC ratio that were determined separately for individuals with eGFR 45–59, 30–44, and < 30 mL/min/1.73 m^2^ are displayed in Table 3. Central 95% reference intervals for kappa FLC, lambda FLC, and FLC ratio were also determined and are demonstrated in Supplementary Table III. Sensitivity analyses of participants with SCr measured at the time of screening yielded similar results displayed in Supplementary Tables IVA–C.

**Table 3:** Novel reference intervals for serum kappa FLC (mg/L), serum lambda FLC (mg/L), and FLC ratio in individuals with eGFR of 45–59, 30–44, and < 30 mL/min/1.73 m^2^.

Group	0.5th percentile		99.5th percentile	
	Value	95% CI	Value	95% CI
Kappa (mg/L)				
eGFR 45–59	7.8	7.6–8.2	83.6	70.2–96.2
eGFR 30–44	8.8	6.3–10.4	103.3	86.9–165.1
eGFR < 30	11.7	11.1–17.0	265.1	133.4–304.2
Lambda (mg/L)				
eGFR 45–59	7.3	6.5–7.6	65.1	52.6–73.2
eGFR 30–44	8.2	6.9–9.3	73.2	66.9–93.3
eGFR < 30	12.6	10.2–14.1	150.9	115.5–173.4
FLC ratio				
eGFR 45–59	0.46	0.41–0.51	2.62	2.24–6.93
eGFR 30–44	0.48	0.37–0.54	3.38	2.45–12.3
eGFR < 30	0.54	0.53–0.61	3.30	2.27–10.9

Central 99% reference intervals. *eGFR* Estimated glomerular filtration rate, *FLC* Free light chain, *CI* Confidence interval.

### FLC and FLC ratio in participants treated for end-stage kidney disease

Of the 42 participants receiving RRT for end-stage kidney disease at the time of screening and therefore analysed seperately were ten receiving dialysis, of whom eight were on hemodialysis and two on peritoneal dialysis, and 32 had a functioning kidney graft. Participants on dialysis had a significantly higher median (IQR) serum kappa FLC level of 89.0 mg/L (67.2–137.2; *p* = 0.002) and lambda FLC of 66.9 mg/L (57.9–91.6; *p* = 0.004), than those with eGFR 30–59 mL/min/1.73 m^2^ or eGFR < 30 mL/min/1.73 m^2^ (kappa FLC, *p* = 0.02; lambda FLC, *p* = 0.02). The median (IQR) FLC ratio in participants on dialysis was 1.27 (1.22–1.49), which was similar as in participants with eGFR 45–59 mL/min/1.73 m^2^ (*p* = 0.33) and 30–44 mL/min/1.73 m^2^ (*p* = 0.95). Participants on dialysis also had a similar FLC ratio as participants with eGFR < 30 mL/min/1.73 m^2^ (*p* = 0.37). Both participants on peritoneal dialysis had FLC and FLC ratio within our novel reference interval for eGFR <30 mL/min/1.73 m^2^. Of the eight participants on hemodialysis during screening, none (0%) had abnormally high serum kappa FLC level and two (25%) abnormally high serum lambda FLC level. However, all the participants had a normal FLC ratio, and therefore, no individual fulfilled criteria for LC-MGUS.

Of the 32 participants with functioning kidney transplant at the time of screening, 16 (50%), 13 (41%) and 3 (9%) had eGFR 45–59, 30–44, and < 30 mL/min/1.73 m^2^, respectively. No statistical difference was observed between serum kappa FLC and lambda FLC levels or the FLC ratio in participants with a kidney transplant and the corresponding eGFR subgroups among other participants. There was one (3%) participant with abnormally low kappa FLC results, but none with abnormal kappa FLC or FLC ratio. No participant fulfilled the criteria for LC-MGUS.

### Prevalence of LC-MGUS in individuals with CKD

Using conventional reference intervals for kappa FLC and lambda FLC and previously published kidney reference intervals for FLC ratio, the crude rate of LC-MGUS was 44 (0.7%), of whom 28 (63%) had kappa LC-MGUS and 16 (37%) lambda LC-MGUS. However, employing our novel reference intervals, the crude prevalence of LC-MGUS in participants with eGFR <60 mL/min/1.73 m^2^ decreased significantly to 31 (0.5%, *p* < 0.001), with 19 (61%) having kappa LC-MGUS and 12 (39%) lambda LC-MGUS. Using the novel reference intervals, the prevalence of LC-MGUS decreased in the 45–59 and 30–44 mL/min/1.73 m^2^ eGFR subgroups compared with the prevalence based on standard intervals with 22 (0.5%), and eight (0.5%) participants with eGFR of 45–59 and 30–44 mL/min/1.73 m^2^, respectively (*p* < 0.001; Table 4). The prevalence of participants with eGFR < 30 mL/min/1.73 m^2^ remained similar with 1 (0.3%) in each group. The crude prevalence of LC-MGUS in individuals with eGFR < 60 mL/min/1.73 m^2^ increased significantly with age, yielding prevalence rates of 0.0%, 0.4%, 0.3%, 0.5%, and 0.7% in age groups <50, 50–59, 60–69, 70–79, and 80 years and above, respectively (*p* < 0.001). The crude prevalence rate of LC-MGUS based on the novel central 95% reference interval was higher or 1.2% (Supplementary Table V).

**Table 4:** Comparison of LC-MGUS rates using standard reference intervals and new reference intervals (FLC ratio and kappa, and lambda FLCs) in participants with eGFR < 60 mL/min/1.73 m^2^.

Group	Kappa LC-MGUS	Kappa LC-MGUS	Lambda LC-MGUS	Lambda LC-MGUS	LC-MGUS	LC-MGUS
	Standard	New	Standard	New	Standard	New
eGFR 45–59	19 (0.4%)	13 (0.3%)	13 (0.3%)	9 (0.2%)	32 (0.7%)	22 (0.5%)
eGFR 30–44	8 (0.5%)	5 (0.3%)	3 (0.2%)	3 (0.2%)	11 (0.8%)	8 (0.5%)
eGFR < 30	1 (0.3%)	1 (0.3%)	0 (0%)	0 (0.0%)	1 (0.3%)	1 (0.3%)

*eGFR* Estimated glomerular filtration rate, *LC-MGUS* Light chain monoclonal gammopathy of undetermined significance.

## Discussion

Using data from the population-based, prospective iStopMM screening study, we show that a majority of individuals with CKD and without evidence of monoclonal gammopathy had serum kappa FLC or lambda FLC levels outside current reference intervals. Moreover, while the proportion of participants that had a FLC ratio outside the standard reference interval was too large (9%), the proportion that was outside of the previously published kidney reference interval was low (0.7%). FLC and FLC ratio increased with declining eGFR and increasing age, although age-related increase in FLC levels diminished after taking eGFR into account. We propose new reference intervals for serum kappa and lambda FLC, and the FLC ratio based on kidney function and show that our novel reference intervals performed well in patients on dialysis therapy and in kidney transplant recipients. We suggest adapting these novel kidney reference intervals for assessment of monoclonal gammopathies in individuals with CKD.

Serum kappa and lambda FLC and the FLC ratio increased significantly with worsening kidney function. FLCs are primarily removed by the kidneys and thus kidney function is a major determinant of the serum levels of both kappa and lambda FLCs [23]. Kappa FLCs are formed at approximately twice the rate of lambda FLC in a normal state [24]. Lambda FLCs are more prone to form dimers, increasing their molecular weight and slowing their kidney clearance [7]. With decreasing kidney function, the serum half life of FLCs lengthens which increases the levels of FLCs despite the absence of a pathological plasma cell disorder [7]. This effect is well demonstrated in our study as 9.1% of participants had FLC ratio outside the standard reference interval (0.26–1.65), emphasizing the need for a kidney reference interval. This is comparable to the previous study by Hutchison et al. in a cohort of 688 patients that was used to define the current kidney reference interval for FLC ratio [9]. However, the concentrations of FLCs were much lower in our cohort (median kappa FLC 21.7 vs. 43.8 mg/L and median lambda FLC 19.0 vs. 38.0 mg/L). The reason for discrepancy between the two studies is probably multifactorial and can partly be explained by lower median eGFR (29 vs. 51 mL/min/1.73 m^2^) in their study, and possibly by different methods for estimating GFR.

Using the previously published kidney reference interval for FLC ratio in our cohort yielded a prevalence rate of LC-MGUS of 0.7%. If the previously published kidney reference intervals were reliable, this would suggests that the prevalence of LC-MGUS is not increased in individuals with CKD [5]. Previous information on the prevalence of LC-MGUS in patients with CKD is limited. Dispenzieri *et al*. estimated the prevalence of LC-MGUS in a general cohort as 0.8%, but did not include information on kidney function [5]. Fenton et al reported a prevalence of 1.6%, but information on SPEP and IFE results was only available for a small part of their cohort, causing high rate of undetected heavy chain gammopathies [25], but where SPEP was available they reported an increased rate of heavy chain MGUS in patients with CKD [25]. Furthermore, the prevalence of LC-MGUS increased with age which is in agreement with the results of previous research on MGUS among individuals with preserved kidney function [5, 12]. All patients in our study with abnormal FLC ratio based on the aforementioned kidney reference interval also had abnormal FLCs. This differs from previous data on FLC and FLC ratio in the general population where the majority (63%) of persons with abnormal FLC ratio without heavy chain MGUS have normal FLCs [5]. The definition of LC-MGUS requires abnormal levels of both FLC ratio and the involved FLC [16]. Current reference intervals therefore select a different group of individuals with CKD compared with persons with normal kidney function. This indicates that the previously published kidney FLC ratio interval is relatively wide compared to the kappa FLC and lambda FLC reference intervals, resulting in skewed assessment of monoclonal disorders in CKD patients. This notion is important as previous studies on MGUS follow-up, progression, and outcomes are mostly based on data from individuals with normal kidney function [5, 26].

When we used our novel kidney reference intervals, the prevalence of LC-MGUS was 0.5%. The changes in reference intervals therefore caused a relative decrease in the prevalence of LC-MGUS among participants with CKD of 29% compared with previously published kidney reference intervals. The prevalence was lower than previously reported in a general population [5]. This is based on our novel central 99% reference interval, but when the novel central 95% reference interval was used the prevalence increased substantially to 1.2%. We recommend the use of the central 99% reference interval instead of 95% reference interval in clinical practice to decrease the false positive rate of LC-MGUS.

Importantly, in our cohort both kappa and lambda FLC increased with age, but when stratified by eGFR the effect of age diminished, indicating that age-related increment of FLC levels reflects the decline in kidney function that occurs with aging. This is comparable to a previous study showing that age-related increase in FLC diminished when divided with serum cystatin C levels [8]. Participants on dialysis at the time of screening had higher absolute levels of kappa and lambda FLC than those with eGFR < 30 mL/min/1.73 m^2^, while their FLC ratio was similar. Dialysis treatment lowers the serum kappa FLC level relatively more than the serum lambda FLC level, causing a lowering of the FLC ratio [27]. An accurate assessment of monoclonal gammopathies in kidney transplant patients is very important as the risk of lymphoproliferative disorders is increased in this population [28]. In our study, the levels of FLCs and FLC ratio in kidney transplant recipients was comparable to other participants with similar eGFR, suggesting that our novel reference intervals perform well in this subgroup. This is in line with a previous study showing that the level of FLCs is not affected by immunosuppressive therapy but rather by graft function [29].

The clinical implications of our findings are substantial. We propose that the novel reference intervals be implemented in clinical practice when assessing serum FLCs in individuals with CKD stage 3 and above. These reference intervals should be used with caution in individuals with acute kidney injury as the SCr and eGFR do not reflect baseline kidney function, and a repeated FLC measurement when kidney function has stabilized is therefore recommended.

A major strength of this study is the large scope of high-quality data. The nationwide population-based cohort comprising 51% of all Icelanders born 1975 or earlier who were screened with SPEP, IFE, and FLC, with all previous SCr measurements available for all participants, allowed us to carry out a meticulous examination of monoclonal gammopathies and accurate assessment of kidney function. All SPEP, IFE, and FLC measurements were performed at the same laboratory using the same methods, thereby decreasing the effect of inter-assay variability. The size of the cohort allowed division of reference intervals into subgroups based on kidney function, yielding more precise determination of the intervals. Furthermore, this study was carried out in a screened population and thus free from selection bias present in other studies.

A limitation of the study is that SCr measurements were not always performed on the same day as the FLC measurements. However, the effect of this shortcoming was decreased by excluding SCr values from more than than one year before the FLC screening as well as measurements during an episode of acute kidney injury detected by a validated algorithm. Additionally, sensitivity analyses of participants‘ SCr levels measured at the time of screening (72% of total cohort) were performed. Information on urine protein electrophoresis, urine immunofixation, and bone marrow biopsy would have complemented the dataset in our study. The group with eGFR < 30 mL/min/1.73 m^2^ comprised relatively few participants causing the accuracy of the central 99% reference interval determination to be less reliable compared to the other eGFR groups. The iStopMM study currently does not include extensive data on outcomes or information on how effectively these reference intervals reflect true presence of monoclonality. Mass spectrometry has been shown to be a reliable method for detecting monoclonal proteins and should be used in future studies to validate the reference intervals. There were few participants in our study dialysis and with kidney transplant patients, making interpretation of our results difficult in that subgroup. In addition, we did not have information on the exact timing of hemodialysis sessions with regards to time of screening. Finally, there is genetic and ethnic homogeneity in the study population, therefore possible issues with generalization.

In conclusion, using data from the iStopMM study comprising a large population-based screened cohort with careful assessment of monoclonal gammopathies and kidney function, we established that current reference intervals for FLC and FLC ratio are inaccurate. Furthermore, we propose new more reliable kidney reference intervals for serum kappa and lambda FLC, and the FLC ratio based on the level of kidney function. Our findings show that these reference intervals also appear to be accurate in individuals on dialysis and in kidney transplant recipients. The novel reference intervals for absolute FLCs are wider and higher than the standard intervals. Furthermore, we suggest narrowing the kidney reference interval for FLC ratio. Implementing the novel reference intervals in clinical practice is likely to increase the utility of the assay for monoclonal gammopathies in individuals with CKD.

## Supplementary information

Supplementary information

## Data Availability

The datasets generated during and/or analysed during the current study are available from the corresponding author on reasonable request. The code generated and used during the current study is available from the corresponding author on reasonable request.
