# Supplementary material for: Defining new reference intervals for serum free light chains in individuals with chronic kidney disease: Results of the iStopMM study
Source: Blood Cancer J. 2022 Sep 14;12(9):133. doi: 10.1038/s41408-022-00732-3 (PMC9470548; doi:10.1038/s41408-022-00732-3)
Supplement: Supplementary file 1 — Supplementary information [file 41408_2022_732_MOESM1_ESM.docx]

Defining New Reference Intervals for Serum Free Light Chains in Individuals With Chronic Kidney Disease: Results of the iStopMM Study

**Supplementary information**

Supplementary Tables:

Supplementary Table IA. Central 95 percentiles and 95% confidence intervals for serum kappa FLC assessed in all participants with eGFR <60 mL/min/1.73 m^2^. Rate of abnormal results in subgroups of sex, age, and level of reduced eGFR when a single unified whole group reference is utilized.

| Group | 2.5th percentile | | 97.5th percentile | | Proportion % | |
| --- | --- | --- | --- | --- | --- | --- |
|  | Value | 95% CI | Value | 95% CI | < 2.5th | > 97.5th |
| All individuals | 10.4 | 10.1 – 10.5 | 66.9 | 63.7 – 71.0 | 2.5% | 2.5% |
| Male | 10.9 | 10.6 – 11.4 | 74.6 | 67.8 – 79.9 | 1.9% | 3.5% |
| Female | 10.2 | 9.9 – 10.4 | 58.6 | 54.5 – 62.6 | 3.0% | 1.7% |
| Age category |  |  |  |  |  |  |
| **≤**70 | 9.4 | 8.9 – 9.8 | 55.9 | 49.6 – 59.7 | 4.8% | 1.5% |
| > 70 | 11.2 | 10.9 – 11.4 | 70.3 | 65.6 – 74.0 | 1.5% | 2.9% |
| eGFR category |  |  |  |  |  |  |
| 45-59 | 10.0 | 9.8 – 10.1 | 47.0 | 44.8 – 49.2 | 3.2% | 0.9% |
| 30-44 | 12.5 | 11.8 – 13.3 | 70.8 | 65.9 – 76.2 | 0.9% | 3.1% |
| 15-29 | 17.5 | 15.5 – 19.0 | 103.1 | 94.7 – 117.0 | 0.0% | 13.7% |
| < 15 | 23.1 | 10.2 – 25.1 | 236.2 | 210.6 – 345.4 | 0.0% | 54.5% |

Abbreviations: eGFR, estimated glomerular filtration rate; CI, confidence interval

Supplementary Table IB. Central 95 percentiles and 95% confidence intervals for serum lambda FLC assessed in all participants with eGFR < 60 mL/min/1.73 m^2^. Rate of abnormal results in subgroups of sex, age, and level of reduced eGFR when a single unified whole group reference is utilized.

| Group | 2.5th percentile | | 97.5th percentile | | Proportion % | |
| --- | --- | --- | --- | --- | --- | --- |
|  | Value | 95% CI | Value | 95% CI | < 2.5th | > 97.5th |
| All individuals | 9.5 | 9.3 – 9.7 | 49.7 | 47.5 – 51.3 | 2.5% | 2.5% |
| Male | 9.7 | 9.5 – 10.0 | 54.6 | 51.3 – 58.8 | 2.1% | 3.2% |
| Female | 9.3 | 9.0 – 9.5 | 47.2 | 45.6 – 49.8 | 2.9% | 1.9% |
| Age category |  |  |  |  |  |  |
| **≤**70 | 9.0 | 8.6 – 9.4 | 48.8 | 45.7 – 52.4 | 3.8% | 2.3% |
| > 70 | 9.8 | 9.6 – 10.0 | 49.9 | 46.8 – 51.7 | 2.0% | 2.5% |
| eGFR category |  |  |  |  |  |  |
| 45-59 | 9.1 | 8.9 – 9.5 | 39.2 | 37.0 – 40.8 | 3.3% | 0.9% |
| 30-44 | 10.9 | 10.1 – 11.8 | 52.8 | 49.3 – 55.8 | 1.0% | 3.4% |
| 15-29 | 14.4 | 13.5 – 15.5 | 80.9 | 70.8 – 90.6 | 0.0% | 12.5% |
| < 15 | 18.6 | 7.9 – 21.0 | 163.7 | 153.9 – 215.0 | 0.0% | 49.1% |

Abbreviations: eGFR, estimated glomerular filtration rate; CI, confidence interval.

Supplementary Table IC. Central 95 percentiles and 95% confidence intervals for serum FLC ratio assessed in all participants with eGFR < 60 mL/min/1.73 m^2^. Rate of abnormal results in subgroups of sex, age, and level of reduced eGFR when a single unified whole group reference is utilized.

| Group | 2.5th percentile | | 97.5th percentile | | Proportion % | |
| --- | --- | --- | --- | --- | --- | --- |
|  | Value | 95% CI | Value | 95% CI | < 2.5th | > 97.5th |
| All individuals | 0.64 | 0.63 – 0.66 | 1.98 | 1.93 – 2.01 | 2.5% | 2.5% |
| Male | 0.68 | 0.66 – 0.70 | 2.03 | 2.00 – 2.09 | 1.7% | 2.9% |
| Female | 0.63 | 0.61 – 0.64 | 1.95 | 1.91 – 2.00 | 2.9% | 2.1% |
| Age category |  |  |  |  |  |  |
| **≤**70 | 0.64 | 0.62 – 0.67 | 1.87 | 1.80 – 1.96 | 2.6% | 1.5% |
| > 70 | 0.65 | 0.63 – 0.67 | 2.03 | 2.00 – 2.08 | 2.3% | 2.9% |
| eGFR category |  |  |  |  |  |  |
| 45-59 | 0.63 | 0.62 – 0.65 | 1.92 | 1.87 – 1.96 | 2.6% | 1.9% |
| 30-44 | 0.68 | 0.65 – 0.71 | 2.06 | 1.91 – 2.15 | 1.6% | 3.3% |
| 15-29 | 0.67 | 0.57 – 0.73 | 2.19 | 2.02 – 2.33 | 1.5% | 7.0% |
| < 15 | 0.61 | 0.43 – 0.61 | 2.07 | 2.03 – 2.28 | 3.6% | 3.6% |

Abbreviations: eGFR, estimated glomerular filtration rate; CI, confidence interval.

Supplementary Table II. Spearman correlation of kappa and lambda FLC and FLC ratio with age in eGFR subgroups.

| eGFR  (mL/min/1.73m^2^) | N | Kappa | | Lambda | | FLC ratio | |
| --- | --- | --- | --- | --- | --- | --- | --- |
|  |  | Correlation | p value | Correlation | p value | Correlation | p value |
| 45 – 59 | 4612 | 0.23 | <0.001 | 0.14 | <0.001 | 0.14 | <0.001 |
| 30 – 44 | 1465 | 0.10 | <0.001 | 0.01 | 0.786 | 0.16 | <0.001 |
| 15 – 29 | 329 | 0.01 | 0.905 | -0.10 | 0.074 | 0.14 | 0.012 |
| < 15 | 55 | 0.01 | 0.958 | -0.18 | 0.340 | 0.39 | 0.035 |

Abbreviations: eGFR, estimated glomerular filtration rate; FLC, free light chains.

Supplementary Table III. Novel central 95% reference intervals for serum kappa FLC (mg/L), serum lambda FLC (mg/L), and FLC ratio in individuals with eGFR of 45-59, 30-44, and < 30 mL/min/1.73 m^2^

| Kappa (mg/L) |  | | | |
| --- | --- | --- | --- | --- |
| Group | 2.5th percentile | | 97.5th percentile | |
|  | Value | 95% CI | Value | 95% CI |
| eGFR 45 – 59 | 10.0 | 9.8 – 10.1 | 47.0 | 44.8 – 49.2 |
| eGFR 30 – 44 | 12.5 | 11.8 – 13.3 | 70.8 | 65.9 – 76.2 |
| eGFR < 30 | 17.8 | 15.7 – 19.3 | 117.4 | 94.3 – 130.4 |
| Lambda (mg/L) |  | |  | |
| Group | 2.5th percentile | | 97.5th percentile | |
|  | Value | 95% CI | Value | 95% CI |
| eGFR 45-59 | 9.1 | 8.9 – 9.5 | 39.2 | 37.0 – 40.8 |
| eGFR 30 – 44 | 10.9 | 10.1 – 11.8 | 52.8 | 49.3 – 55.8 |
| eGFR < 30 | 14.5 | 13.8 – 15.2 | 94.7 | 68.4 – 103.9 |
| FLC ratio |  | |  | |
| Group | 2.5th percentile | | 97.5th percentile | |
|  | Value | 95% CI | Value | 95% CI |
| eGFR 45-59 | 0.63 | 0.62 – 0.65 | 1.92 | 1.87 – 1.96 |
| eGFR 30 – 44 | 0.68 | 0.65 – 0.71 | 2.06 | 1.91 – 2.15 |
| eGFR < 30 | 0.67 | 0.57 – 0.72 | 2.17 | 2.03 – 2.30 |

Abbreviations: eGFR, estimated glomerular filtration rate; FLC, free light chain; CI, confidence interval

Supplementary Table IVA. Sensitivity analysis based only on participants with SCr measured on the same day as screening. Reference intervals for serum kappa FLC (mg/L) in individuals with eGFR of 45-59, 30-44, and < 30 mL/min/1.73 m^2^

| Group | 2.5th percentile | | 97.5th percentile | |
| --- | --- | --- | --- | --- |
|  | Value | 95% CI | Value | 95% CI |
| eGFR 45 – 59 | 10.2 | 10.0 – 10.6 | 46.6 | 44.6 – 48.7 |
| eGFR 30 – 44 | 12.7 | 11.8 – 13.7 | 71.9 | 67.5– 76.9 |
| eGFR < 30 | 18.5 | 15.9 – 20.8 | 122.0 | 92.1 – 139.3 |

Abbreviations: eGFR, estimated glomerular filtration rate; CI, confidence interval.

Supplementary Table IVB. Sensitivity analysis based only on participants with SCr measured on the same day as screening. Novel reference intervals for serum lambda FLC (mg/L) in individuals with eGFR of 45-59, 30-44, and < 30 mL/min/1.73 m^2^

| Group | 2.5th percentile | | 97.5th percentile | |
| --- | --- | --- | --- | --- |
|  | Value | 95% CI | Value | 95% CI |
| eGFR 45-59 | 9.2 | 8.9 – 9.5 | 40.5 | 37.7 – 43.1 |
| eGFR 30 – 44 | 10.2 | 8.9 – 10.6 | 53.2 | 48.4 – 55.7 |
| eGFR < 30 | 14.5 | 13.2 – 15.6 | 96.6 | 71.1 – 108.3 |

Abbreviations: eGFR, estimated glomerular filtration rate; CI, confidence interval.

Supplementary Table IVC. Sensitivity analysis based only on participants with serum creatinine measured on the same day as screening. Novel reference intervals for FLC ratio in individuals with eGFR of 45-59, 30-44, and <30 mL/min/1.73 m^2^

| Group | 2.5th percentile | | 97.5th percentile | |
| --- | --- | --- | --- | --- |
|  | Value | 95% CI | Value | 95% CI |
| eGFR 45-59 | 0.66 | 0.64 – 0.67 | 1.92 | 1.87 – 1.97 |
| eGFR 30 – 44 | 0.69 | 0.66 – 0.72 | 2.05 | 1.87 – 2.15 |
| eGFR < 30 | 0.65 | 0.54 – 0.70 | 2.17 | 1.84 – 2.30 |

Abbreviations: eGFR, estimated glomerular filtration rate; FLC, free light chain; CI, confidence interval.

Supplementary Table V. Comparison of LC-MGUS rates using standard reference intervals and new central 95 reference intervals (FLC ratio and kappa, and lambda FLCs) in participants with eGFR < 60 mL/min/1.73 m^2^.

| Group | Kappa LC-MGUS | Kappa LC-MGUS | Lambda LC-MGUS | Lambda LC-MGUS | LC-MGUS | LC-MGUS |
| --- | --- | --- | --- | --- | --- | --- |
|  | Standard | New | Standard | New | Standard | New |
| eGFR 45-59 | 19 (0.4%) | 31 (0.7%) | 13 (0.3%) | 23 (0.5%) | 32 (0.7%) | 54 (1.2%) |
| eGFR 30-44 | 8 (0.5%) | 10 (0.7%) | 3 (0.2%) | 8 (0.5%) | 11 (0.8%) | 18 (1.2%) |
| eGFR < 30 | 1 (0.3%) | 2 (0.5%) | 0 (0%) | 1 (0.3%) | 1 (0.3%) | 3 (0.8%) |

Abbreviations: eGFR, estimated glomerular filtration rate; LC-MGUS, light chain monoclonal gammopathy of undetermined significance.

Supplementary Figures


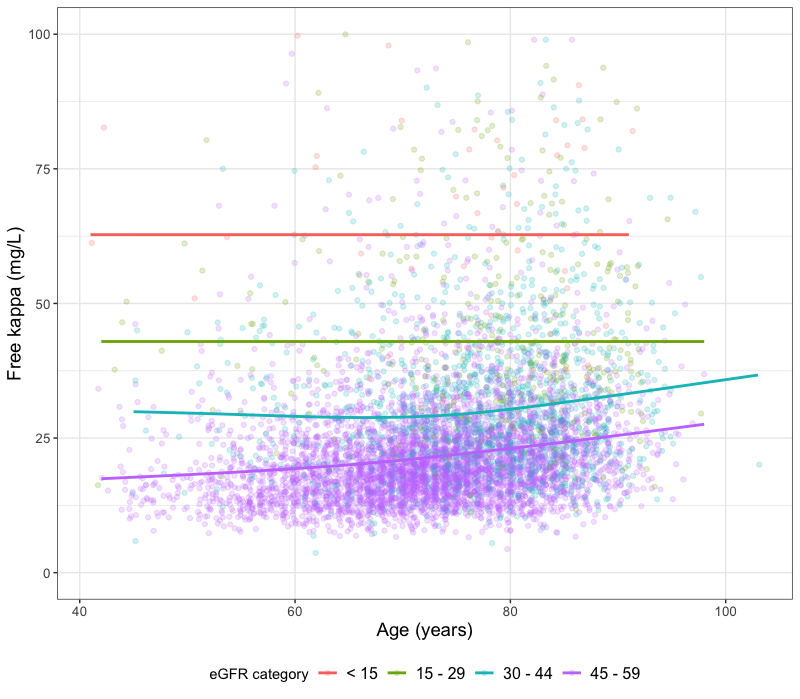


Supplementary Figure 1A. Scatterplot demonstrating correlation between age and serum kappa FLC stratified by level of eGFR at the time of screening.

Abbreviations: eGFR, estimated glomerular filtration rate; FLC, free light chain.


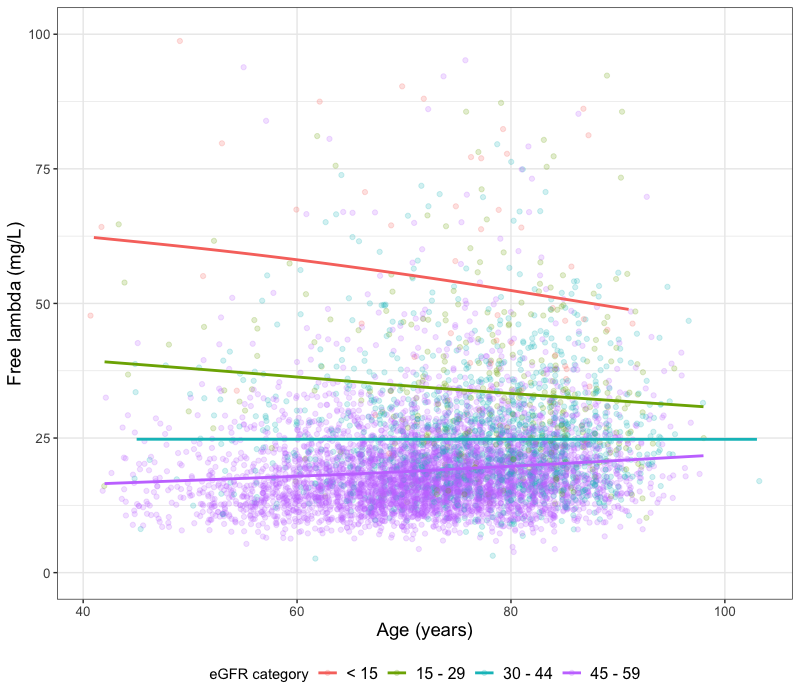


Supplementary Figure 1B. Scatterplot demonstrating correlation between age and serum lambda FLC stratified by level of eGFR at the time of screening.

Abbreviations: eGFR, estimated glomerular filtration rate; FLC, free light chain.


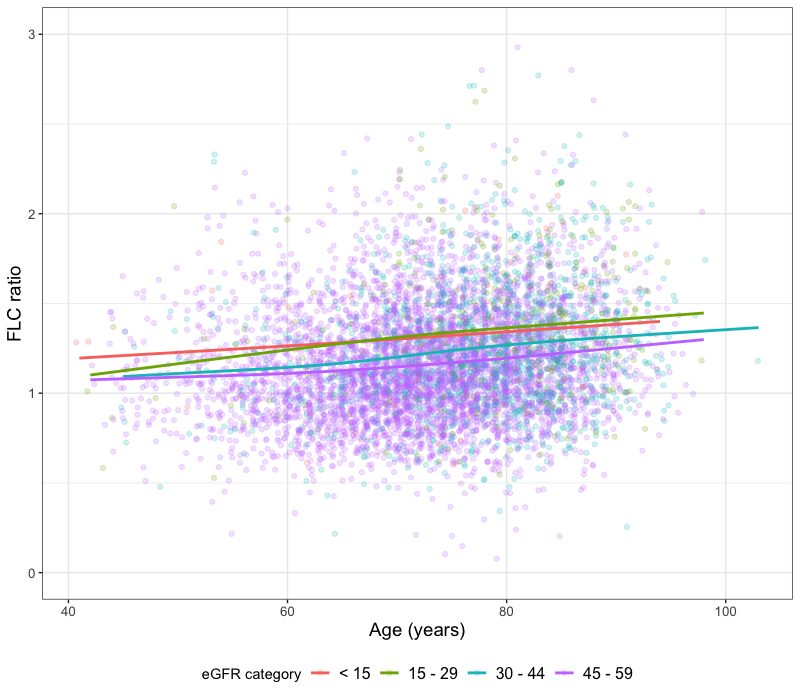


Supplementary Figure 1C

Scatterplot demonstrating correlation between age and serum FLC ratio stratified by level of eGFR at the time of screening.

Abbreviations: eGFR, estimated glomerular filtration rate; FLC, free light chain.
